# Supplementary material for: Assessing Exchange-Correlation Functionals for Heterogeneous Catalysis of Nitrogen Species
Source: J Phys Chem C Nanomater Interfaces. 2024 Jul 1;128(27):11159–75. doi: 10.1021/acs.jpcc.4c01497 (PMC11247500; doi:10.1021/acs.jpcc.4c01497)
Supplement: Supplementary file 1 — jp4c01497_si_001.pdf [file jp4c01497_si_001.pdf]

Assessing Exchange-Correlation Functionals for Heterogeneous Catalysis of Nitrogen Species

Honghui Kim,[†,¶] Neung-Kyung Yu,[‡] Nianhan Tian,[‡] and Andrew J. Medford[*,‡]

† *Department of Chemical and Biomolecular Engineering (BK21 four), Korea Advanced Institute of Science and Technology (KAIST), Daejeon 34141, Republic of Korea*

‡ *School of Chemical & Biomolecular Engineering, Georgia Institute of Technology, Atlanta, Georgia 30332, United States*

¶ *Visiting researcher at School of Chemical & Biomolecular Engineering, Georgia Institute of Technology*

E-mail: ajm@gatech.edu

Computational Details

DFT Details

Initial Position of Adsorbates For Cu(100) and Pd(111), we have two experimental adsorption energies reported, Cu(100)+NH_3 and Pd(111)+NO.[1] We adopted the optimized configuration of the experimental adsorption data to be an initial binding configuration for Cu(100) and Pd(111). For rutile TiO_2(110), the five-fold Ti site is chosen.[2] For MoO_3(100), oxygen vacancy site is chosen.[3] In the case of OCUPUY, the open vanadium site is chosen. For MIL−125, placement of small gas molecules is less straightforward. We conducted grand-canonical monte carlo simulation of a N_2 molecule and adopted the result as an initial configuration for other adsorbates. However, majority of adsorbates had too weak binding from the initial guess, which requires additional manipulation of the position by intuition. After all these processes, we were able to set initial position near the oxygen atoms bound to the metal node or the connection between the metal node and the dicarboxylate linker.

Correction on Experimental Unitcell Volume As we compare unitcell volume from DFT to one from experiment, we need to subtract the contribution from thermal expansion and zero-point effects to compare DFT-calculated volume directly to the experiment. The amount of volume change from the two factors are calculated using eq. (S1) and eq. (S2).[4] $\alpha_{V,rt}$ is the volume thermal expansion coefficient at room temperature, B_0 is the bulk modulus, B_1 is derivative of the bulk modulus, and θ_D is the Debye temperature.

$$\frac{\Delta V^{thermal}}{V} \approx \int_0^{T_{rt}} \alpha_{V,rt} \frac{T}{T_{rt}} \, dT = \frac{\alpha_{V,rt} T_{rt}}{2} \tag{S1}$$

$$\Delta V^{ZPE} = \frac{(B_1 - 1)\zeta}{2B_0} = \frac{9}{16}(B_1 - 1)\frac{k_B \Theta_D}{B_0} \tag{S2}$$

We used corrected volume of Cu and Pd from the previous benchmark from Wellendorff et al.[5] For TiO_2 and MoO_3, we used eq. (S1) and eq. (S2). $\alpha_{V,rt}$, B_0, B_1 , and θ_D are from mulitple experimental papers.[6–11] For MOFs, we were not able to find a full set of experimentally measured properties for correcting the volume, so the correction scheme is not applied to MOFs.

RPA Details

RPA Calculation In RPA@PBE approach for metallic systems, the finite-temperature RPA formalism[12] was utilized with Fermi smearing and a smearing width (σ) of 0.05 eV. For non-metallic systems, the non-finite-temperature formalism and low σ values ($\leq$ 0.005 eV) were used. In RPA@PBEx calculations, which utilize global hybrid functionals such as PBE0 and PBEx50, the non-finite-temperature formalism and Gaussian smearing were applied with smearing widths of 0.05 eV for metals and 0.005 −0.01 eV for non-metals. Generally, RPA@PBE and RPA@HSE06 calculations are more unstable and sensitive to the calculation parameters, such as smearing widths and k-point meshes, compared to RPA@PBEx approaches.

RPA molecular Energy Observations indicate that the absolute contributions of $E_{c,RPA}$ to both molecular atomization energy and formation energy are underestimated in RPA calculations. To address this issue, a scaling factor was multiplied to $E_{c,RPA}$. The scaling factor was optimized to minimize the error in atomization energy of molecules considered in Figure S1, compared to CCSD(T) values. CCSD(T) energies were calculated using ORCA v5.0.4,[13] and the extrapolation of basis sets was applied using aug-cc-pVQZ and aug-cc-pV5Z. Unlike formation energy, atomization energy is less affected by error cancellation between the energies of reactants and products, making it a more suitable dataset for fitting purposes. Utilizing the PBE-optimized molecular structures, the scaling factors was determined to be 1.17 for the optRPA@PBEx50 approach. The error of optRPA@PBEx50

Table S1: Three sets of different kspacing and kinetic energy cutoff used for testing convergence of energy in six solid materials.

	A1	A2	A3
KSPACING ($\mathrm{\AA}^{-1}$)	0.5	0.4	0.3
ENCUT (eV)	600	600	700

Table S2: Γ-centered k-point mesh employed to evaluate energy convergence in solid materials.

Accuracy setup	Cu	Pd	TiO_2	MoO_3	OCUPUY	MIL−125
A1	$4 \times 4 \times 4$	$4 \times 4 \times 4$	$3 \times 3 \times 5$	$4 \times 4 \times 1$	$2 \times 2 \times 2$	$1 \times 1 \times 1$
A2	$5 \times 5 \times 5$	$5 \times 5 \times 5$	$4 \times 4 \times 6$	$5 \times 5 \times 2$	$3 \times 2 \times 2$	$2 \times 2 \times 2$
A3	$6 \times 6 \times 6$	$6 \times 6 \times 6$	$5 \times 5 \times 8$	$6 \times 6 \times 2$	$3 \times 3 \times 2$	$2 \times 2 \times 2$

Table S3: Convergence test result of bulk materials' energy in eV/atom unit. One GGA (PBE-D3), one meta-GGA (SCAN-D3) and one hybrid functional (PBE0-D3) are chosen for the test. The most accurate setup (A3) is set to be zero, so the numbers to be the energy difference from the A3 setup. The convergence criterion is $0.025\,\mathrm{eV/atom}$.

	PBE-D3			SCAN-D3			PBE0-D3		
	A1	A2	A3	A1	A2	A3	A1	A2	A3
Cu (4 atoms)	−0.0033	0.0318	0	−0.004	0.0305	0	−0.0275	0.0243	0
Pd (4 atoms)	0.0308	0.0038	0	0.0463	0.0125	0	0.0193	−0.0193	0
TiO_2 (6 atoms)	0.0028	0.0023	0	0.0025	0.002	0	−0.0053	0.0002	0
MoO_3 (16 atoms)	0.0035	0.0024	0	0.003	0.002	0	−0.0046	0.002	0
OCUPUY (56 atoms)	0.0023	−0.002	0	−0.0072	−0.0021	0	0.0009		0
MIL-125 (116 atoms)	0.0031	0.0032	0	0.0034	0.0034	0	−0.0018	0.0037	0

is comparable to the numerical error in CCSD(T), as can be seen in the error of CCSD(T)-F12, a CCSD(T) method with F12 correction, which improves the basis set dependency.[14] The same scaling approach would not work for RPA@PBE as we have seen examples where the signs of E_{HF} and $E_{c,RPA}$ of molecules were not correct. In the assessment of the sensitivity of the $E_{c,RPA}$ scaling factor using k-fold cross-validation, we observed consistent values for the scaling factor in different fold configurations. For the optRPA@PBEx50 approach, considering k=3, 5, and 10 folds, the mean (median, standard deviation) of the optimized scaling factors were found to be 1.168 (1.168, 0.0002), 1.167 (1.168, 0.0010), and 1.167 (1.168, 0.0007), respectively. The scaling factor optimized based on the formation energy was identified as 1.174. When employing B3LYP-optimized structures and atomization energies, and the scaling factors were determined to be 1.176 for optRPA@PBEx50 and 1.248 for optRPA@PBEx75. B3LYP-optimized structures were used to obtain the energies for Figure S1 and Figure S2, but deviations when PBE geometries are used were observed to be minimal in all cases tested.

RPA Energy Convergence Detailed convergence test results are provided for RPA calculations across various systems. RPA calculations are computationally intensive, especially in terms of memory requirements. Therefore, a lower kinetic energy cutoff (ENCUT) of 400 eV was chosen for calculating $\Delta_r \mathrm{E}_{c,ads}$ as defined below, which represents the $\mathrm{E}_{c,RPA}$ contribution to the adsorption energy $\Delta_r \mathrm{E}_{ads}$, after a convergence test (Figure S3).

$$\Delta_r E_{ads} = E_{solid+gas} - E_{solid} - E_{gas} \tag{S3}$$

Among the GW pseudopotentials used, the oxygen (O_GW_new) and nitrogen (N_GW_new) pseudopotentials have the highest default ENCUTs of 434.4 and 420.9 eV. Consequently, the observation that, for MoO_3 systems containing N-containing adsorbates, the differences in $\Delta_r \mathrm{E}_{ads}$ between ENCUTs of 400 and 500 eV are less than 0.02 eV suggests that 400 eV is a reasonable choice (Figure S3(a)). Although differences of about 0.05 eV are observed

Table S4: Convergence test result of gas molecules' energy in eV unit. One GGA (PBE-D3), one meta-GGA (SCAN-D3) and one hybrid functional (PBE0-D3) are chosen for the test. Only Γ point is sammpled, and the kinetic energy cutoff of 400 eV, 500 eV, 600 eV, 700 eV are tested. The most accurate setup (700 eV) is set to be zero, so the numbers to be the energy difference. The convergence criterion is 0.025 eV, but to be aligned with the convergence test of bulk materials, 600 eV is chosen for the calculations of gas formation energy and gas reaction energy.

	PBE-D3				SCAN-D3				PBE0-D3			
	400 eV	500 eV	600 eV	700 eV	400 eV	500 eV	600 eV	700 eV	400 eV	500 eV	600 eV	700 eV
N_2	0.051	0.015	0.006	0	0.112	0.015	0.002	0	0.071	0.014	0.004	0
O_2	0.030	0.012	0.006	0	0.026	0.013	0.007	0	0.018	0.013	0.007	0

Table S5: Parameters used for D3 correction.

	RPBE	PBE	SCAN	PBE0	HSE06	B3LYP
s_8	0.8318	0.7875	0.0000	1.2177	2.3100	1.9889
a_1	0.1820	0.4289	0.5380	0.4145	0.3830	0.3981
a_2	4.0094	4.4407	5.4200	4.8593	5.6850	4.4211

Table S6: Convergence test results of RPA molecular atomization energy in eV unit. The most accurate setup (700 eV and 600 eV for E_{HF} and $E_{c,RPA}$, respectively) is set to be zero, so the numbers to be the energy difference.

	RPA@PBE0			
	600/400 eV	600/500 eV	600/600 eV	700/600 eV
N_2	0.098	0.023	0.002	0
O_2	0.028	0.024	0.028	0

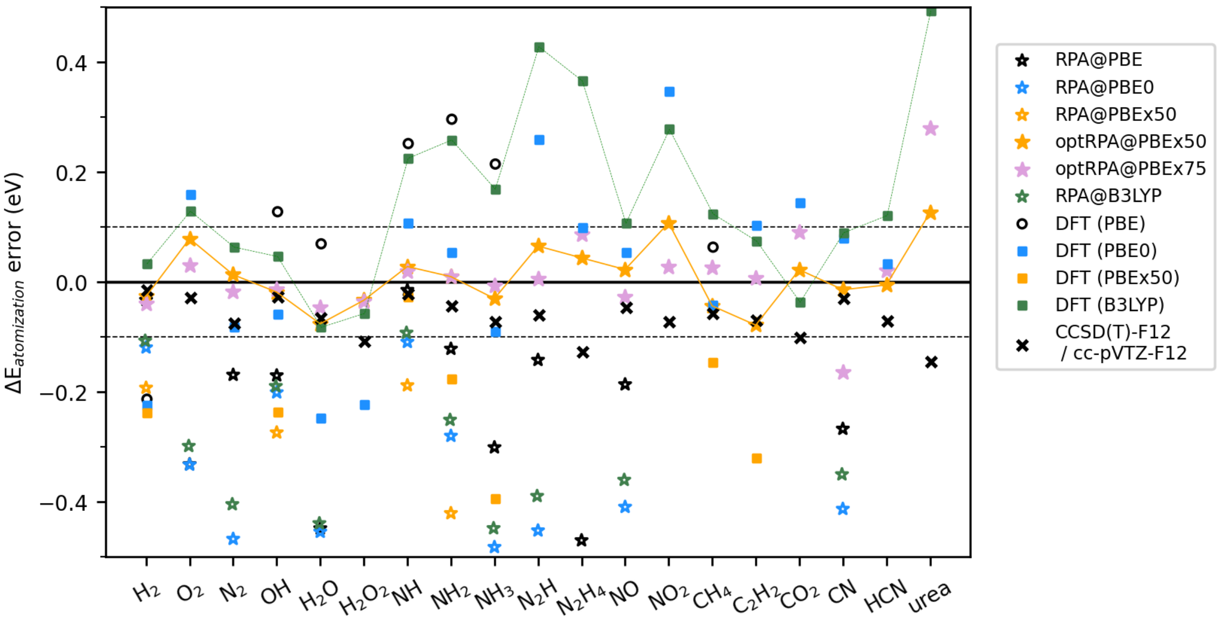

Figure S1: Error of the atomization energies from CCSD(T) values.

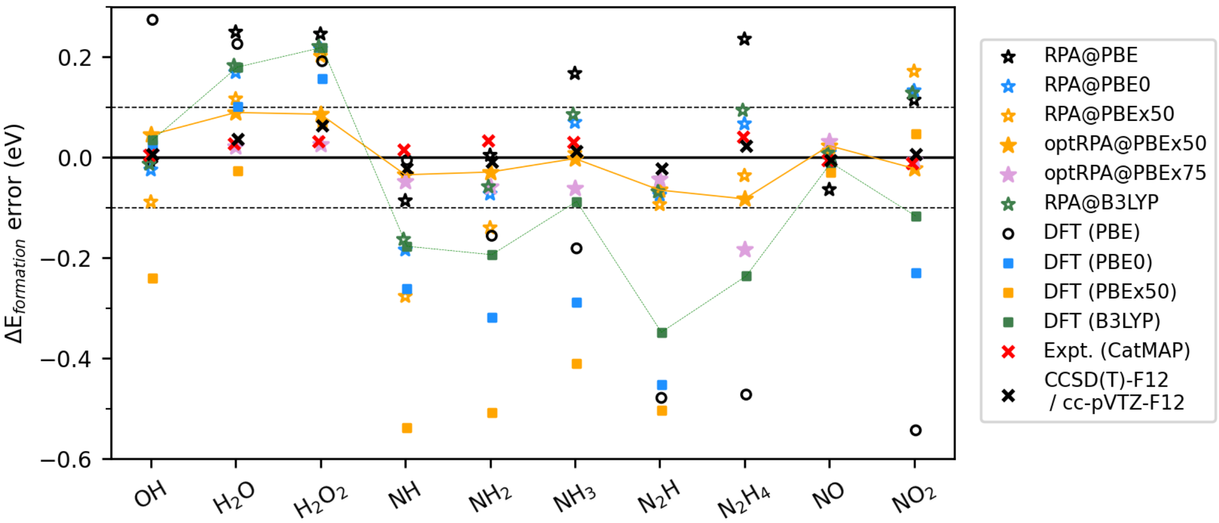

Figure S2: Error of the formation energies from CCSD(T) values.

for OCUPUY, the absolute $\Delta_r E_{c,ads}$ exceeds 5 eV, corresponding to roughly a 1% error (Figure S3(b)).

In the RPA@PBE approach to metallic systems, the non-finite-temperature formalism may break down; thus, the finite-temperature formalism should be used. Additionally, the calculations on metallic systems are sensitive to σ, as the metallic slabs, with and without adsorbates, respond differently to variations in σ (Figure S4). The different responses can be somewhat alleviated by subtracting half of the electronic entropy term from E_{HF}, similar to how the zero-temperature energy is obtained in DFT calculation. Furthermore, a correction to E_{HF} related to partial occupancies[15] was not applied, as it worsens the energy convergence in terms of σ (Figure S4(a)). According to the VASP forum, the correction cannot be rigorously derived within the finite-temperature formalism. Therefore, the correction to E_{HF} was applied only to calculations using the non-finite-temperature formalism. RPA calculations on metallic systems generally require much denser k-point grids than standard DFT. For the metallic slabs of Cu and Pd, k-point grids of 7×7×7 and 12×12×12 were used, respectively (See Figure S4(b) and Figure S5(b) for energy convergence test). Another parameter worth discussing is the choice of GW pseudopotentials. Specifically, for Cu, a GW pseudopotential with fewer valence electrons was utilized after convergence test (Figure S5(a)). The reason behind the choice is the high computational cost associated with generating PBEx wavefunctions, rather than the actual RPA calculation step to get $E_{c,RPA}$.

RPA@PBE calculations on TiO_2 reveal a sensitivity to σ of non-metallic systems when adsorbate-induced states are near the Fermi level, akin to observations in the metallic systems. Likewise, the sensitivity to σ occurs due to the different responses of slabs, with and without adsorbates, to changes in σ (Figure S6(a)). To minimize the smearing effect, extremely low σ values ($\leq$ 0.005 eV) and the non-finite-temperature formalism were used (Figure S6(b)). However, it is important to note that such low σ values can lead to numerical instability. For the RPA methods prone to instability, such as RPA@PBE and RPA@HSE06, it is important to ensure numerical stability by verifying the consistency of DFT energy be-

fore and after the exact diagonalization step. Additionally, the RPA@PBE method requires denser k-point grids compared to those required by global hybrid-based approach, likely due to the influence of states near the Fermi level (Figure S6(b,c)). In RPA@PBEx approaches for non-metallic systems, the same or denser k-point grids, compared to the ones used for DFT calculations, were employed, except for TiO_2, where its energy convergence is tested in Figure S6(c).

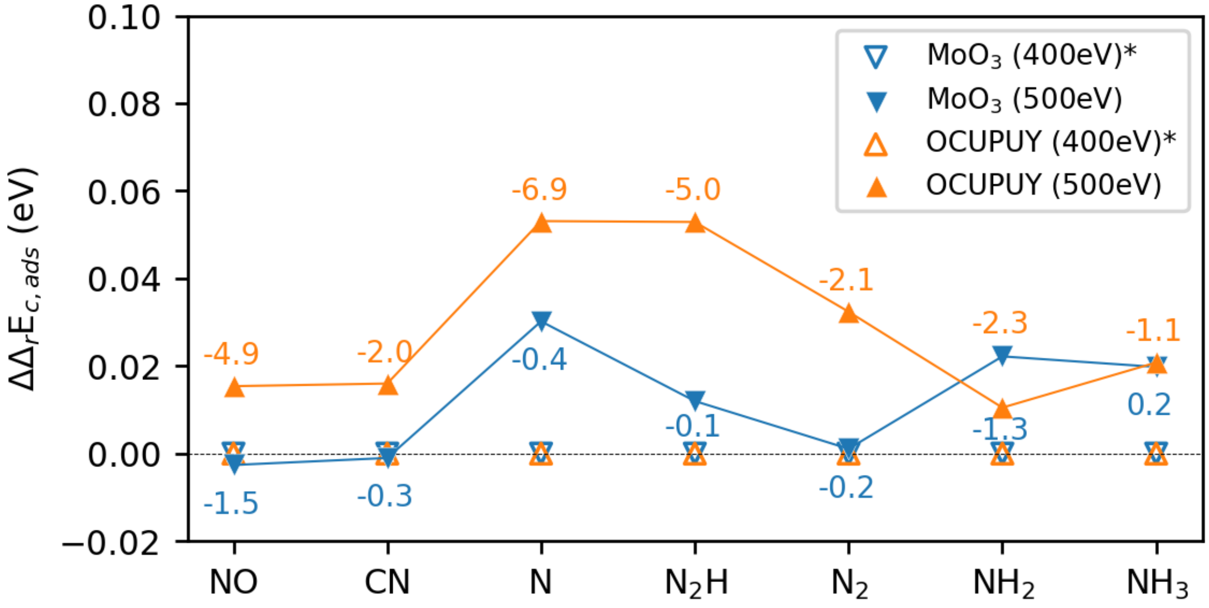

Figure S3: Convergence test results of $E_{c,RPA}$ contribution to $\Delta_r E_{ads}$ in terms of ECUT. *denotes the parameters used in the main text.

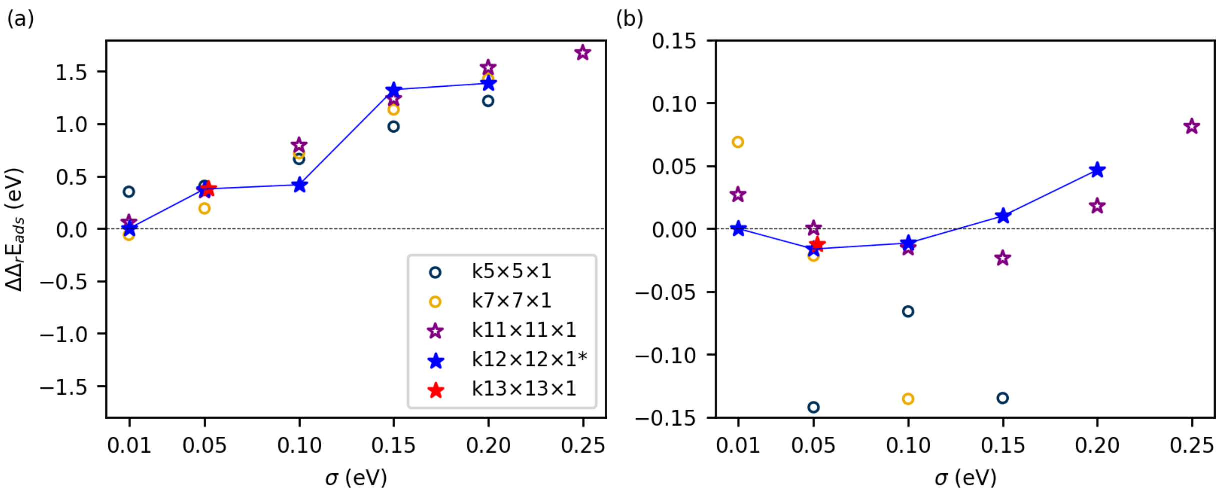

Figure S4: Convergence test of RPA@PBE calculations on Pd. (a) with E_{HF} correction. (b) without E_{HF} correction and after subtracting $\frac{1}{2}T\Delta S_{elec}$. * denotes the parameters used in the main text.

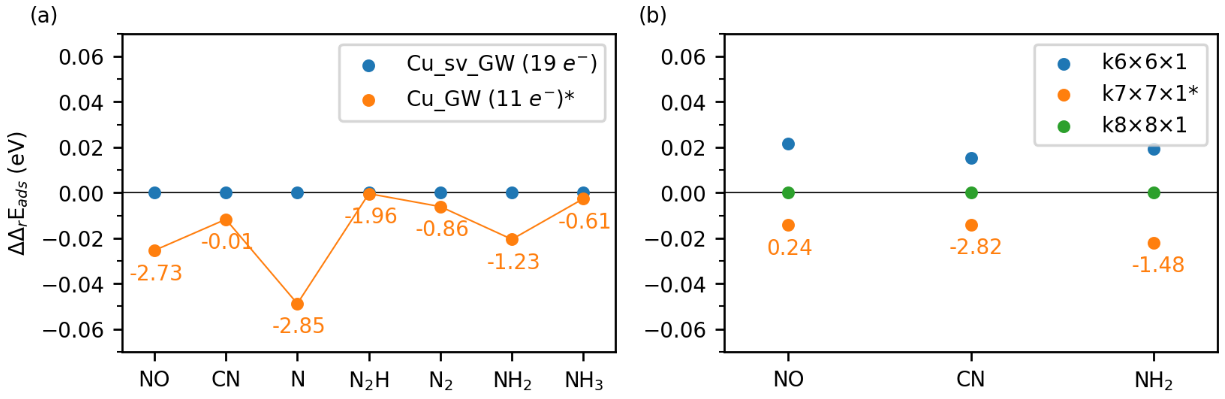

Figure S5: Convergence test results of RPA@PBE0 calculations on Cu in terms of pseudopotentials (a) and k-points grid (b). * denotes the parameter used in the main text.

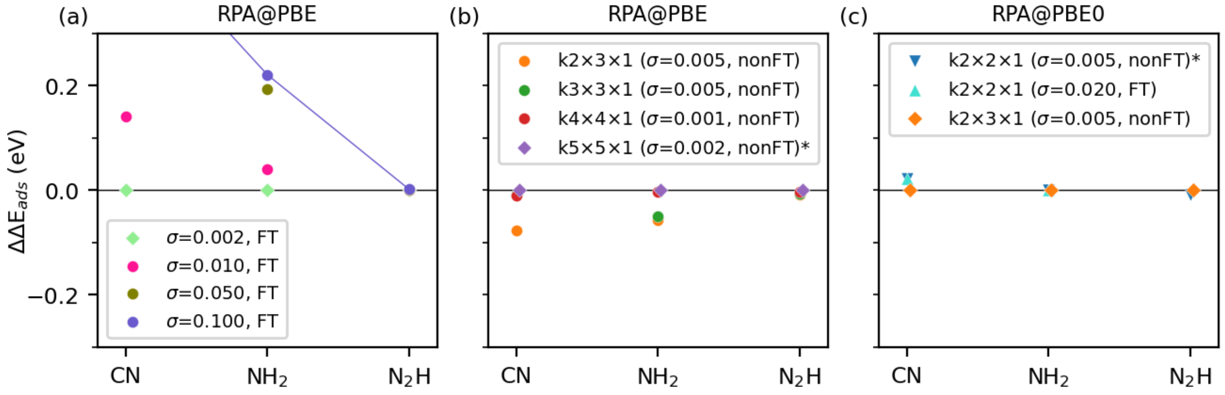

Figure S6: Convergence test results of RPA calculations on TiO_2. (a) RPA@PBE with finite-temperature (FT) formalism. (b) RPA@PBE without finite-temperature formalism. (c) RPA@PBE0. * deontes the parameter used in the main text.

Supplemenatry Figures and Tables

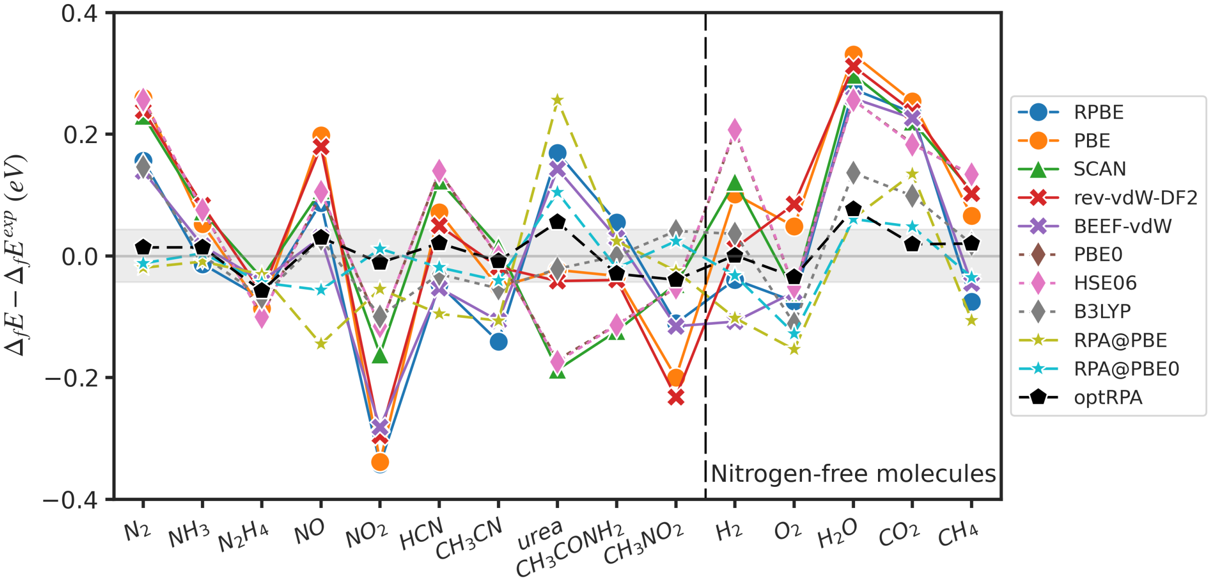

Figure S7: Error of calculated formation energy per each gas molecule without D3 correction.

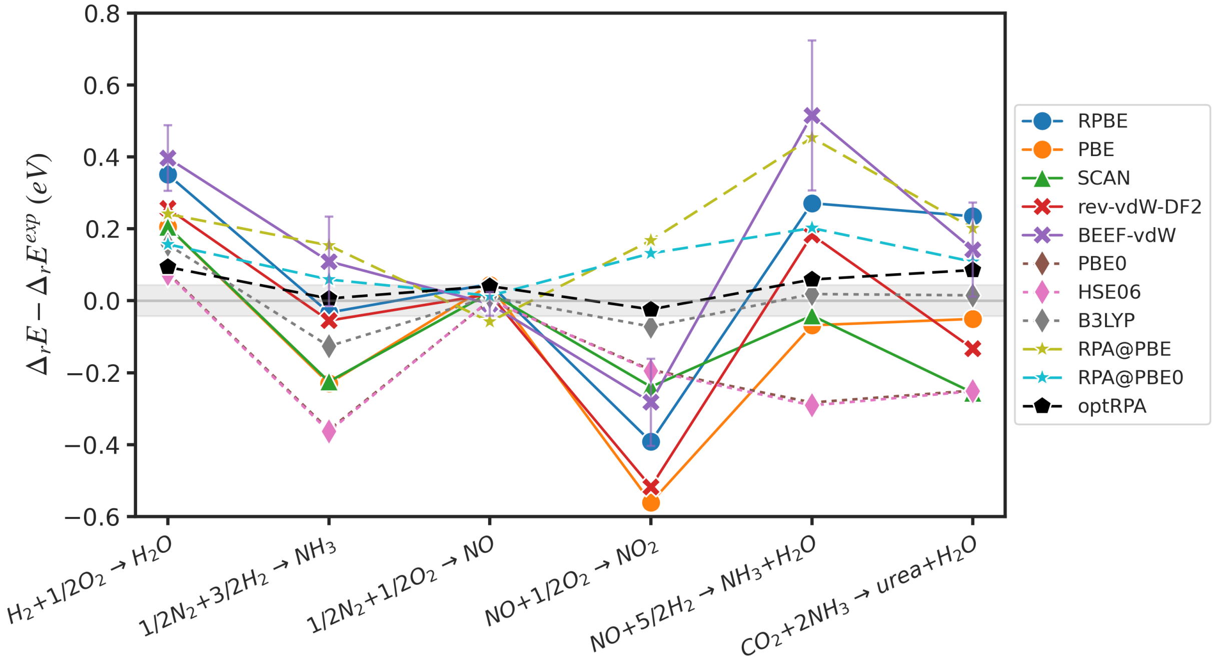

Figure S8: Error of calculated reaction energy from experimental reaction energy without D3 correction.

Table S7: Error of the calculated formation energies and reaction energies compared to the experimental formation and reaction energies without D3 correction.

	$\Delta_f^{LS}E - \Delta_f E^{exp}$			$\Delta_r E - \Delta_r E^{exp}$		
	MaxError	MAE	RMSE	MaxError	MAE	RMSE
RPBE	−0.342	0.126	0.156	−0.392	0.221	0.261
PBE	−0.339	0.141	0.177	−0.561	0.193	0.264
SCAN	0.298	0.127	0.149	−0.256	0.165	0.191
rev-vdW-DF2	0.312	0.133	0.167	−0.518	0.194	0.254
BEEF-vdW	−0.282	0.110	0.138	0.515	0.242	0.299
PBE0	0.256	0.130	0.148	−0.359	0.194	0.229
HSE06	0.256	0.131	0.149	−0.363	0.196	0.232
B3LYP	0.146	0.059	0.075	0.156	0.066	0.088
RPA@PBE	0.256	0.088	0.110	0.453	0.213	0.245
RPA@PBE0	−0.128	0.043	0.054	0.202	0.112	0.128
optRPA	0.077	0.029	0.035	0.094	0.051	0.060

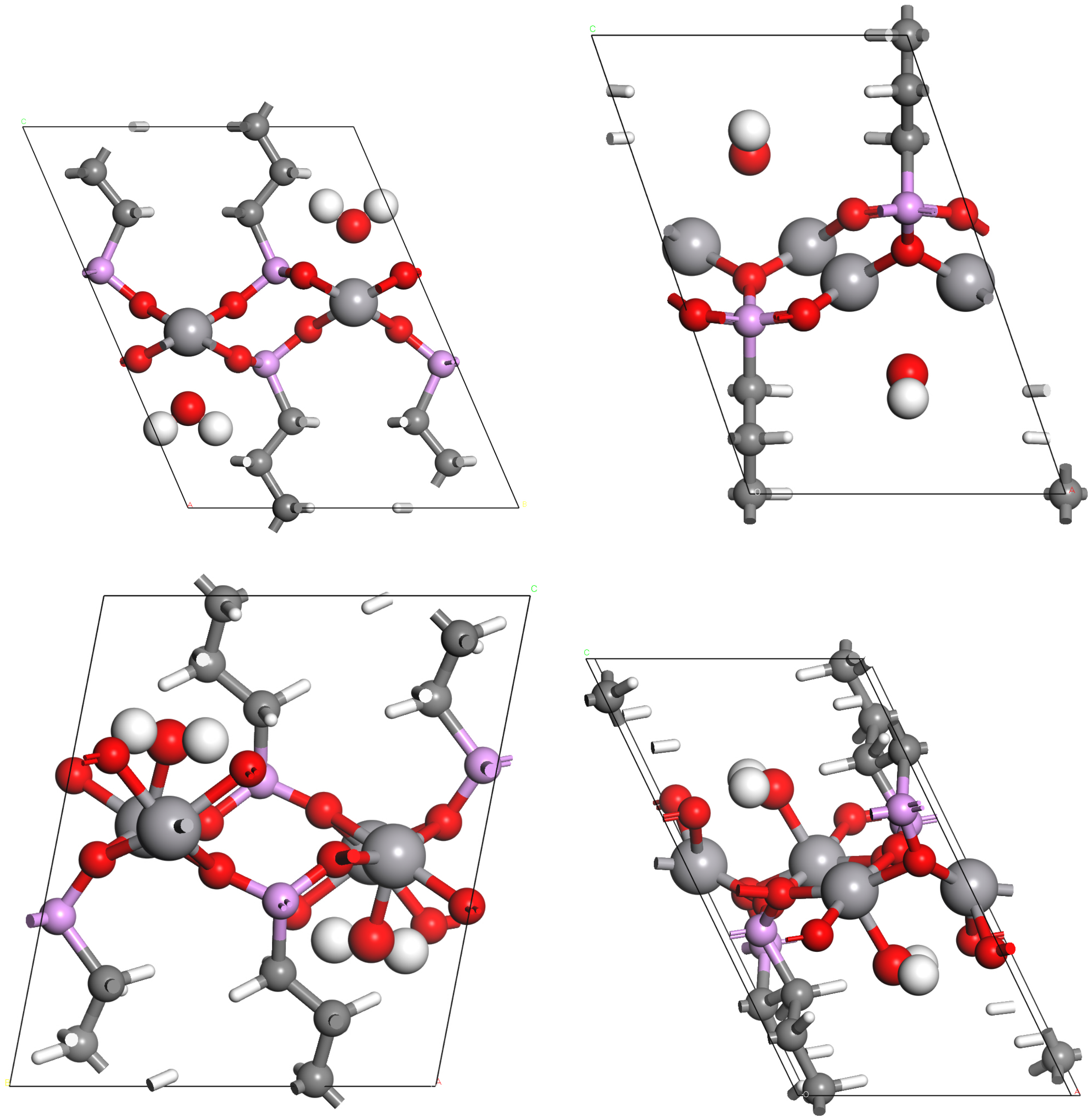

Figure S9: Images of relaxed OCUPUY unitcell using (top) PBE-D3 and (bottom) SCAN-D3. (color code: light grey=V, grey=C, red=O, white=H, purple=P; vanadium atoms and water molecules are magnified in the images.)

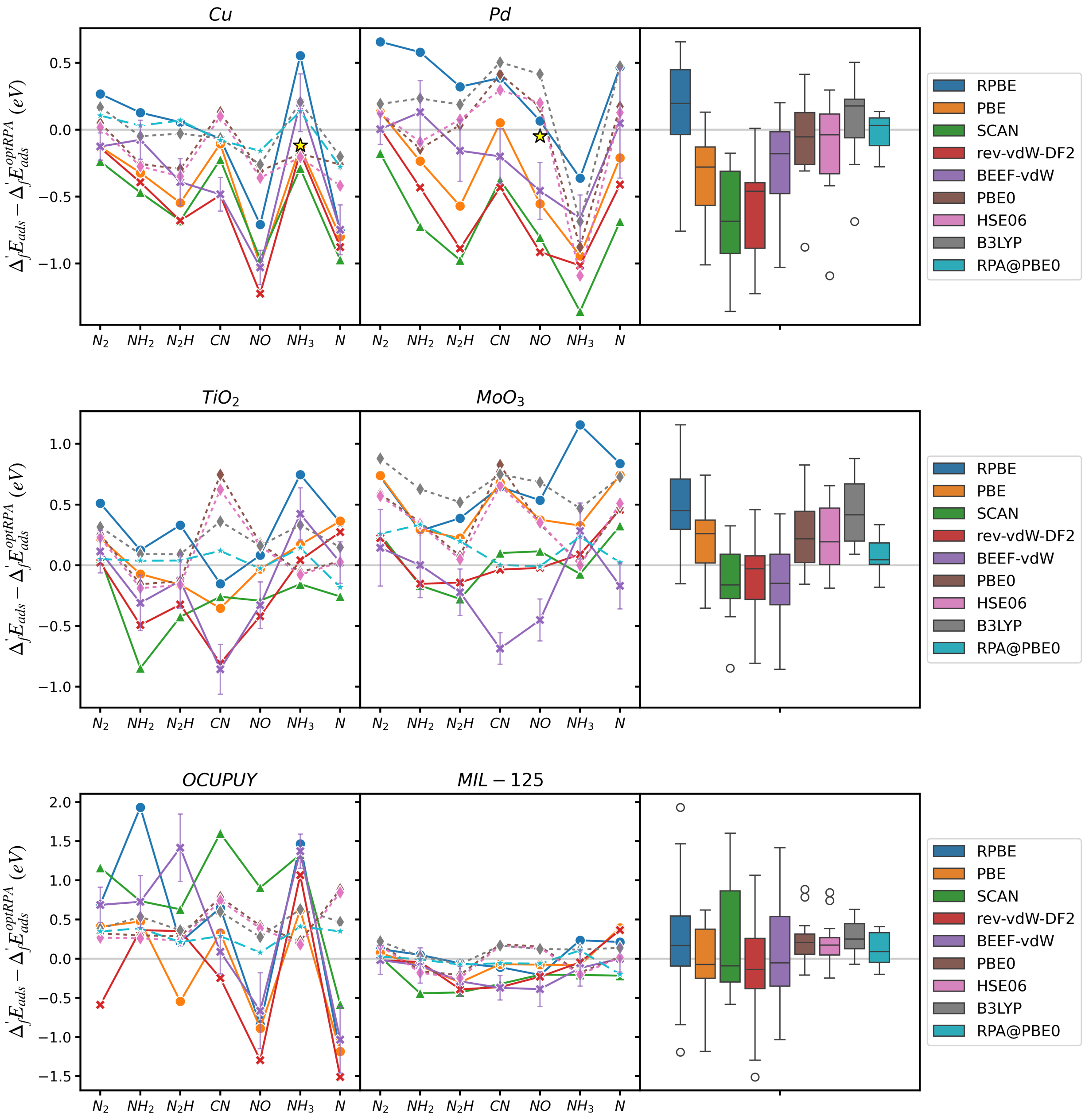

Figure S10: Error of the calculated adsorption energies without D3 correction in (top) metals, (center) metal oxides, and (bottom) MOFs compared to the adsorption energy of optRPA. Each point is drawn by subtracting D3 correction in Figure 4. Distribution of errors for each functional are plotted on the right as box and whisker plots.

Table S8: Error of calculated unitcell volume per atom from the corrected experimental volume with and without D3 correction. Unit in Å^3/atom.

	$V_{DFT} - V_{exp}$ with D3	$V_{DFT} - V_{exp}$ without D3
	PBE	
Cu	-0.264	0.430
Pd	0.087	0.832
TiO_2	0.248	0.438
MoO_3	0.701	2.215
	RPBE	
MoO_3	-0.181	3.288

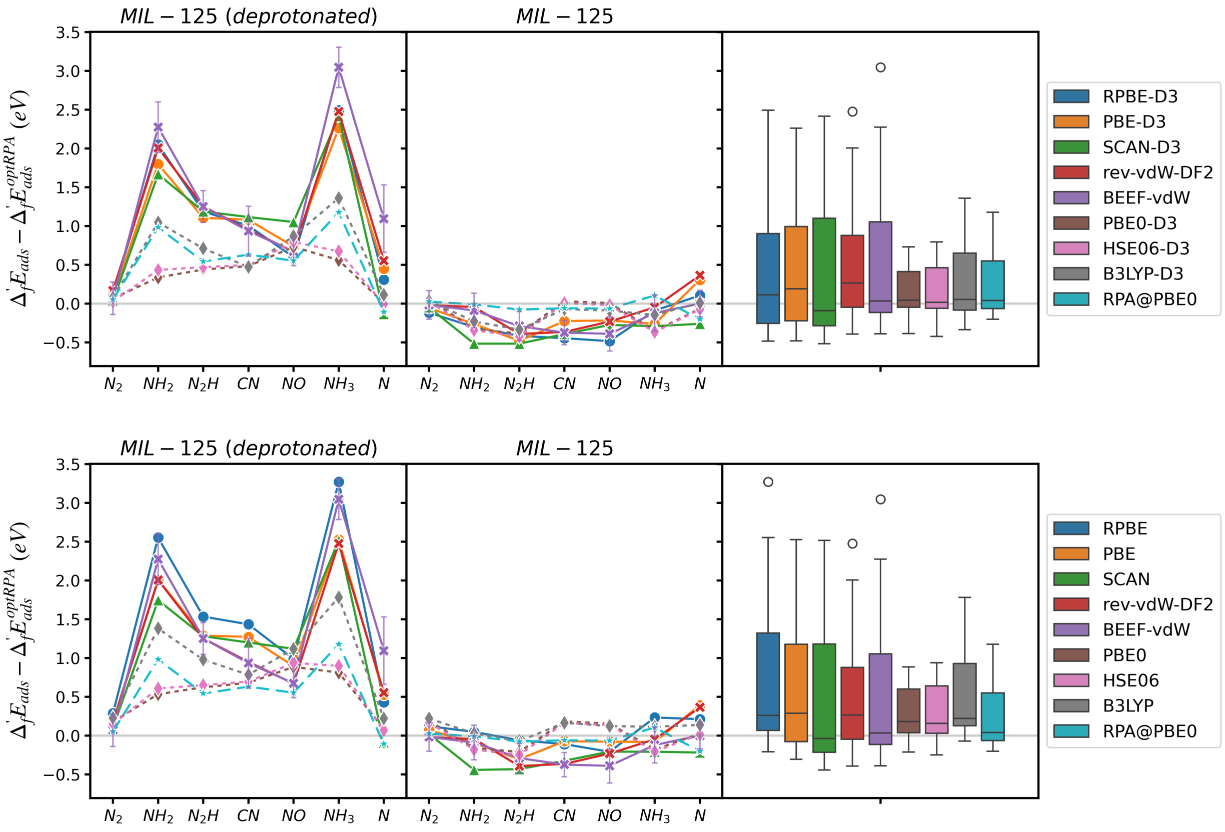

Figure S11: Error of the calculated adsorption energies with (top) and without (bottom) D3 correction compared to the adsorption energy of optRPA in deprotonated MIL-125 and protonated MIL-125.

Table S9: Calculated adsorption energy ($\Delta_f^{'} E_{i,ads}$) from RPAs (optRPA, RPA@PBEx50, RPA@PBE0, RPA@PBE). Unit in eV.

		$\Delta_f^{'} E_{ads}$			
	adsorbate	optRPA	RPA@PBEx50	RPA@PBE0	RPA@PBE
	N_2	0.044	0.186	0.149	0.206
	NH_2	0.024	0.112	0.054	0.094
	N_2H	2.215	2.369	2.283	2.237
Cu	CN	2.749	2.782	2.670	2.374
	NO	3.580	3.736	3.421	3.134
	NH_3	−1.321	−1.163	−1.184	−1.136
	N	4.158	4.106	3.882	3.593
	N_2				−0.094
	NH_2				−0.453
	N_2H				1.181
Pd	CN				2.073
	NO				1.818
	NH_3				−0.184
	N				0.727
	N_2	−0.344	−0.271	−0.293	
	NH_2	1.209	1.160	1.246	1.477
	N_2H	1.841	1.878	1.879	1.940
TiO_2	CN	5.624	6.024	5.743	5.355
	NO	3.369	3.411	3.341	
	NH_3	−2.117	−2.039	−1.973	
	N	4.752	4.400	4.572	
	N_2	−0.777	−0.555	−0.519	
	NH_2	−1.743	−1.381	−1.409	
	N_2H	1.239	1.370	1.436	
MoO_3	CN	2.375	2.362	2.377	
	NO	1.334	1.456	1.326	
	NH_3	−2.760	−2.629	−2.525	
	N	1.305	1.331	1.328	
	N_2	−1.741	−1.195	−1.394	
	NH_2	−2.627	−2.224	−2.239	
	N_2H	−1.601	−0.647	−1.390	
OCUPUY	CN	−0.347	0.091	−0.059	
	NO	0.166	0.894	0.243	
	NH_3	−3.350	−3.030	−2.941	
	N	−0.261	1.037	0.088	
	N_2	−0.406	−0.322	−0.379	
	NH_2	1.273	1.229	1.265	
	N_2H	0.134	−0.063	0.058	
MIL−125	CN	3.787	3.838	3.729	
	NO	3.231	3.269	3.169	
	NH_3	−1.101	−1.026	−0.995	
	N	4.789	4.414	4.589	

Table S10: Error of the calculated adsorption energy without D3 correction compared to the adsorption energy of optRPA.

	Metals			Metal Oxides			MOFs		
	MaxError	MSE	RMSE	MaxError	MSE	RMSE	MaxError	MSE	RMSE
RPBE	−0.758	0.113	0.451	1.157	0.469	0.574	1.932	0.225	0.808
PBE	−1.010	−0.384	0.517	0.743	0.251	0.406	−1.185	−0.068	0.511
SCAN	−1.357	−0.638	0.725	−0.847	−0.136	0.322	1.601	0.284	0.774
rev-vdW-DF2	−1.225	−0.575	0.674	−0.808	−0.092	0.335	−1.510	−0.185	0.667
BEEF-vdW	−1.029	−0.282	0.447	−0.857	−0.155	0.378	1.416	0.094	0.697
PBE0	−0.876	−0.088	0.316	0.825	0.253	0.396	0.886	0.230	0.387
HSE06	−1.090	−0.132	0.370	0.655	0.220	0.357	0.846	0.198	0.364
B3LYP	−0.686	0.078	0.320	0.879	0.439	0.508	0.631	0.284	0.353
RPA@PBE0	−0.277	−0.025	0.143	0.334	0.087	0.157	0.408	0.128	0.233

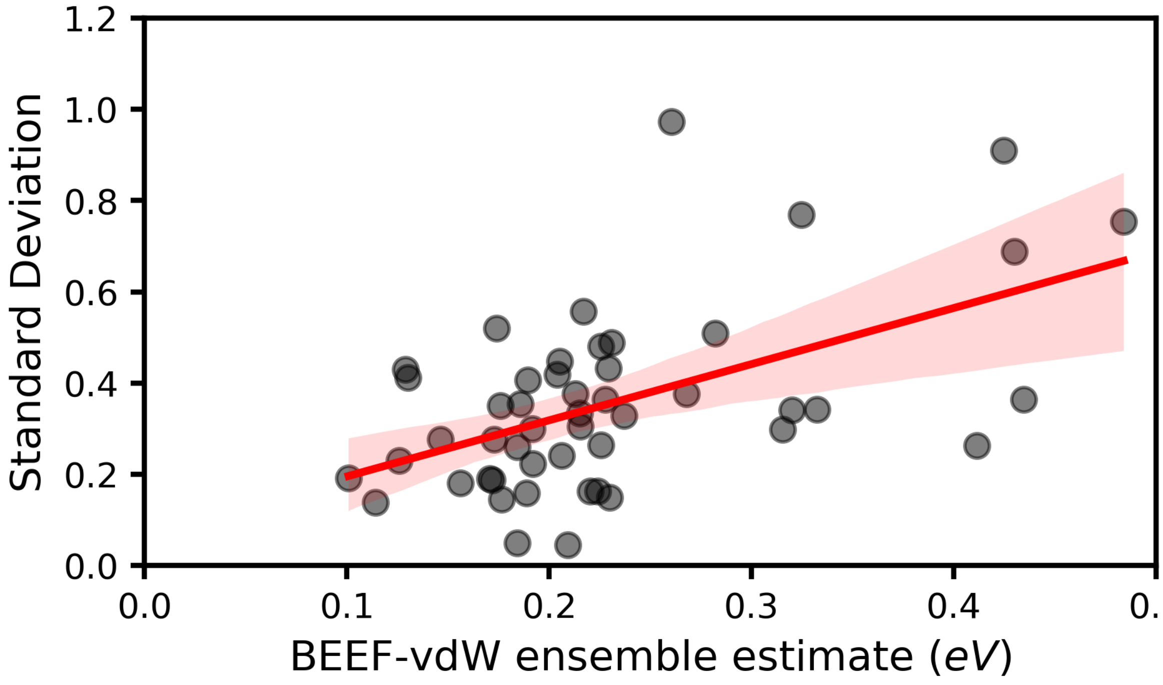

Figure S12: Relation between BEEF-vdW error bar and standard deviation of adsorption energies from different functionals. Rgression line is drawn, and confidence interval of 95% is shaded in red. Pearson correlation coefficient is 0.536.

Table S11: RMSE of the calculated adsorption energy compared to the adsorption energy of optRPA. Unit in eV.

	Cu	Pd	TiO_2	MoO_3	OCUPUY	MIL−125
RPBE-D3	0.951	0.767	0.576	0.568	1.177	0.322
PBE-D3	0.759	0.734	0.328	0.234	0.810	0.283
SCAN-D3	0.725	0.933	0.486	0.214	0.918	0.361
rev-vdW-DF2	0.673	0.676	0.423	0.211	0.907	0.261
BEEF-vdW	0.543	0.323	0.405	0.349	0.957	0.238
PBE0-D3	0.450	0.509	0.325	0.266	0.316	0.235
HSE06-D3	0.512	0.575	0.312	0.281	0.305	0.249
B3LYP-D3	0.460	0.505	0.194	0.368	0.222	0.169
RPA@PBE0	0.143		0.102	0.198	0.315	0.096
RPBE	0.460	0.443	0.396	0.708	1.131	0.159
PBE	0.549	0.484	0.229	0.527	0.694	0.200
SCAN	0.627	0.811	0.404	0.208	1.054	0.297
rev-vdW-DF2	0.673	0.676	0.423	0.211	0.907	0.261
BEEF-vdW	0.543	0.323	0.405	0.349	0.957	0.238
PBE0	0.229	0.384	0.316	0.462	0.521	0.167
HSE06	0.281	0.441	0.276	0.423	0.484	0.173
B3LYP	0.163	0.422	0.240	0.677	0.481	0.136
RPA@PBE0	0.143		0.102	0.198	0.315	0.096

Table S12: Standard deviation and error of the calculated adsorption energy without D3 correction compared to the adsorption energy of optRPA, for each material. "std. dev" stands for standard deviation between functionals (except for RPA results).

	std. dev	MSE	RMSE
Cu	0.253	−0.301	0.476
Pd	0.376	−0.176	0.521
TiO_2	0.307	0.003	0.345
MoO_3	0.336	0.310	0.480
OCUPUY	0.615	0.322	0.818
MIL−125	0.166	−0.056	0.210
Total	0.369	0.017	0.510

Table S13: Deviation of calculated adsorption energies from experimental values (in eV), with and without D3 correction.

	Cu(100)+NH_3*		Pd(111)+NO*	
	$\Delta'_f E_{ads}^{+D3} - \Delta'_f E_{ads}^{exp}$	$\Delta'_f E_{ads} - \Delta'_f E_{ads}^{exp}$	$\Delta'_f E_{ads}^{+D3} - \Delta'_f E_{ads}^{exp}$	$\Delta'_f E_{ads} - \Delta'_f E_{ads}^{exp}$
RPBE	−0.245	0.670	−0.788	0.114
PBE	−0.313	−0.014	−0.786	−0.504
SCAN	−0.307	−0.172	−0.878	−0.756
rev-vdW-DF2	−0.052		−0.866	
BEEF-vdW	0.318		−0.409	
PBE0	−0.361	−0.061	−0.070	0.217
HSE06	−0.390	−0.088	−0.044	0.248
B3LYP	−0.137	0.324	−0.001	0.464
RPA@PBE0	0.327		-	
optRPA	0.117		0.049	

Table S14: Comparison of adsorption energy and interaction energy (same gas and solid geometry from the RPBE optimized gas+solid system, and compute single-point calculation to get energies), and the amount of D3 correction added.

	property	single-point	geometry optimization
MoO_3+CN	$\Delta'_f E_{ads}$ (eV)	1.06	1.45
	D3 correction (eV)	−0.57	−1.46
Pd(111)+N_2	$\Delta'_f E_{ads}$ (eV)	−0.34	−0.32
	D3 correction (eV)	−0.91	−0.88
Pd(111)+CN	$\Delta'_f E_{ads}$ (eV)	1.42	1.45
	D3 correction (eV)	−1.12	−1.02

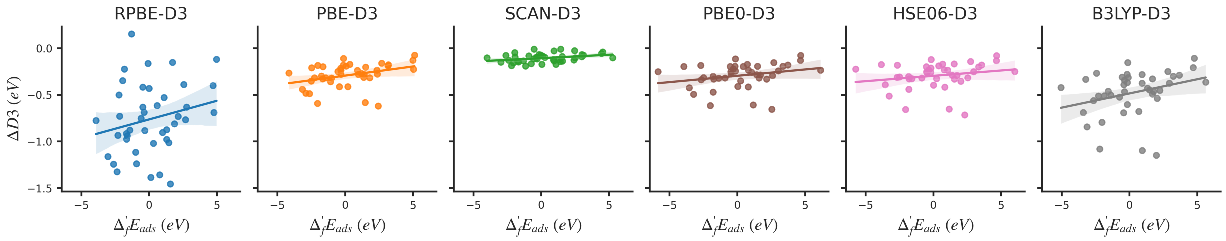

Figure S13: Relation between adsorption energy and D3 correction with regression lines drawn. Confidence interval of 95% is shaded in color.

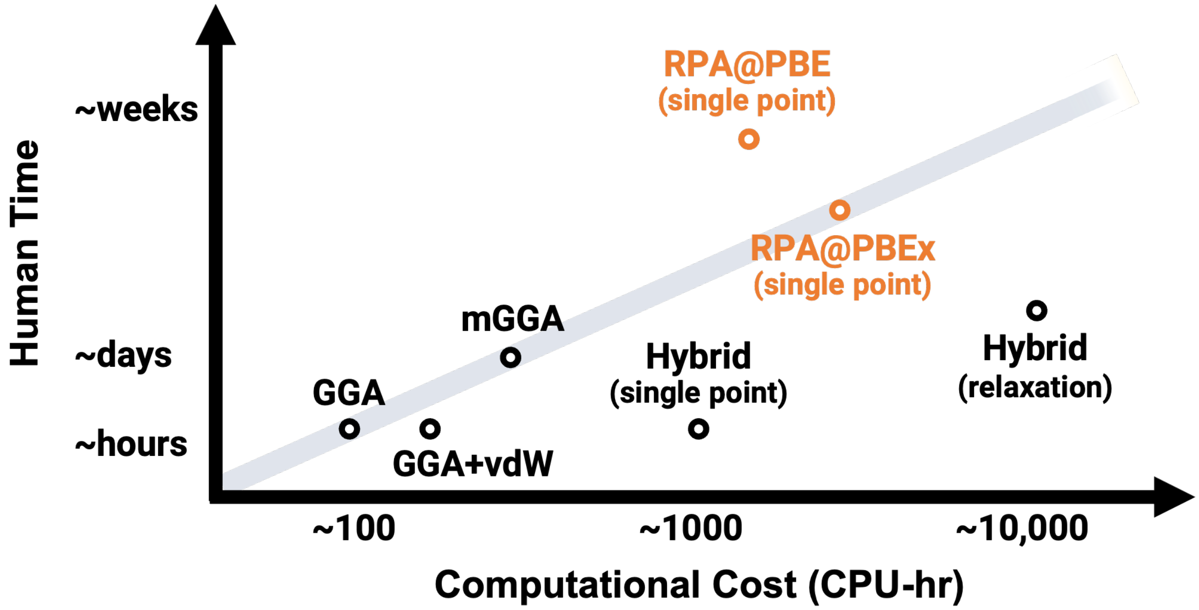

Figure S14: Schematic illustration of computational time and human time required for computing a single adsorption energy for a typical slab system. Human time reflects effort required for convergence testing and post-processing. Representative order-of-magnitude numbers are provided based on our experience in this work. The high memory requirement of RPA is not reflected.

Table S15: Multiple adsorption energies (physisorption and chemisorption) of NO on MoO_3(100) compared to that of optRPA chemisorption. Unit in eV.

	chemisorption	physisorption
RPBE-D3	0.384	1.697
PBE-D3	0.172	1.355
PBE0-D3	0.146	1.666
HSE06-D3	0.184	1.594
B3LYP-D3	0.354	1.273

Table S16: Results of different spin state configurations tested and their energy in OCUPUY, using PBE-D3. Numbers in 'Initial magnetic moments' and 'Optimized magnetic moments' represent magnetic moment of four vanadium atoms in the unit cell. $\bar{3}$ represents -3.

combination of spin states	lowspin	4 up	3 up, 1 down				2 up, 2 down		
Initial magnetic moments	0000	3333	$333\bar{3}$	$33\bar{3}3$	$3\bar{3}33$	$\bar{3}333$	$33\bar{3}\bar{3}$	$3\bar{3}3\bar{3}$	$\bar{3}33\bar{3}$
Optimized magnetic moments	3333	3333	$333\bar{3}$	$33\bar{3}3$	$3\bar{3}33$	$\bar{3}333$	$33\bar{1}\bar{3}$	$3\bar{3}3\bar{3}$	$333\bar{3}$
Energy difference from '3333' (eV)	0.000	0.000	0.053	0.052	0.053	0.052	0.716	0.037	0.053

Table S17: Different spin states and energy after gas adsorbed in OCUPUY, using PBE-D3. Original state represents the result of Figure 4. '3333' state represents the state where all four vanadium atoms in OCUPUY unitcell have up-spin states. ΔE stands for the energy difference of the '3333' state compared to the original state (unit in eV). 'mag' stands for the total magnetic moment. Since NH_2 and CN are already in the '3333' state (or $\bar{3}\bar{3}\bar{3}\bar{3}$), the last row of them is empty.

	N_2*		NH_2*		N_2H*		CN*		NO*		NH_3*		N*	
	ΔE	mag	ΔE	mag	ΔE	mag	ΔE	mag	ΔE	mag	ΔE	mag	ΔE	mag
Original state	0.0	5.7	0.0	−11	0.0	3	0.0	11	0.0	2.4	0.0	6.5	0.0	−0.9
'3333' state	0.210	11.8	-	-	0.113	9.0	-	-	−0.020	9.0	−0.001	12.0	−0.067	9.0

References

(1) Wellendorff, J.; Silbaugh, T. L.; Garcia-Pintos, D.; Nørskov, J. K.; Bligaard, T.; Studt, F.; Campbell, C. T. A benchmark database for adsorption bond energies to transition metal surfaces and comparison to selected DFT functionals. *Surface Science* **2015**, *640*, 36–44.

(2) Comer, B. M.; Medford, A. J. Analysis of Photocatalytic Nitrogen Fixation on Rutile TiO2(110). *ACS Sustainable Chemistry & Engineering* **2018**, *6*, 4648–4660.

(3) Li, Y.; Chen, X.; Zhang, M.; Zhu, Y.; Ren, W.; Mei, Z.; Gu, M.; Pan, F. Oxygen vacancy-rich MoO_{3-x} nanobelts for photocatalytic N2 reduction to NH3 in pure water. *Catalysis Science & Technology* **2019**, *9*, 803–810.

(4) Lejaeghere, K.; Van Speybroeck, V.; Van Oost, G.; Cottenier, S. Error estimates for solid-state density-functional theory predictions: an overview by means of the ground-state elemental crystals. *Critical reviews in solid state and materials sciences* **2014**, *39*, 1–24.

(5) Wellendorff, J.; Lundgaard, K. T.; Møgelhøj, A.; Petzold, V.; Landis, D. D.; Nørskov, J. K.; Bligaard, T.; Jacobsen, K. W. Density functionals for surface science: Exchange-correlation model development with Bayesian error estimation. *Physical Review B* **2012**, *85*, 235149.

(6) Gerward, L.; Staun Olsen, J. Post-rutile high-pressure phases in TiO2. *Journal of Applied Crystallography* **1997**, *30*, 259–264.

(7) Wu, A.; Sladek, R. Elastic Debye temperatures in tetragonal crystals: Their determination and use. *Physical Review B* **1982**, *25*, 5230.

(8) Kirby, R. K. Thermal expansion of rutile from 100 to 700 K. *Journal of Research of the National Bureau of Standards. Section A, Physics and Chemistry* **1967**, *71*, 363.

(9) Yamashita, Y.; Aoki, Y.; Yagi, T.; Jia, J.; Kashiwagi, M.; Oguchi, Y.; Taketoshi, N.; Shigesato, Y. Thermal conductivity across the van der Waals layers of α-MoO3 thin films composed of mosaic domains with in-plane 90 rotations. *Journal of Applied Physics* **2021**, *130*, 085103.

(10) Liu, D.; Lei, W.; Hao, J.; Liu, D.; Liu, B.; Wang, X.; Chen, X.; Cui, Q.; Zou, G.; Liu, J. et al. High-pressure Raman scattering and x-ray diffraction of phase transitions in MoO3. *Journal of applied physics* **2009**, *105*, 023513.

(11) Negishi, H.; Negishi, S.; Kuroiwa, Y.; Sato, N.; Aoyagi, S. Anisotropic thermal expansion of layered MoO 3 crystals. *Physical Review B* **2004**, *69*, 064111.

(12) Kaltak, M.; Klimeš, J.; Kresse, G. Cubic scaling algorithm for the random phase approximation: Self-interstitials and vacancies in Si. *Physical Review B - Condensed Matter and Materials Physics* **2014**, *90*, 054115.

(13) Neese, F. The ORCA program system. *Wiley Interdisciplinary Reviews: Computational Molecular Science* **2012**, *2*, 73–78.

(14) Adler, T. B.; Knizia, G.; Werner, H. J. A simple and efficient CCSD(T)-F12 approximation. *Journal of Chemical Physics* **2007**, *127*, 221106.

(15) Harl, J.; Schimka, L.; Kresse, G. Assessing the quality of the random phase approximation for lattice constants and atomization energies of solids. *Physical Review B - Condensed Matter and Materials Physics* **2010**, *81*, 115126.
